# Actinomycosis, with Report of a Case

**Published:** 1893-01

**Authors:** Roswell Park

**Affiliations:** 510 Delaware Avenue; Buffalo, N. Y.


Buffalo Medical and Surgical Journal

VOL. XXXII. JANUARY, 1893. NO. 6.

### Original Communications.

### ACTINOMYCOSIS, WITH REPORT OF A CASE.[1]

BY ROSWELL PARK, A. M., M. D., Buffalo, N. Y.

UNDER one name or another, the peculiar manifestations now known to be the result of the condition termed actinomycosis, have been recognized in nearly all civilized countries, and have been noticed especially about the head and neck of domestic animals, particularly cattle. The large variety of names given to these lesions constitutes the best indication of the fact that their etiology was unknown. During the past thirty or forty years they have been more and more carefully studied by veterinarians. In England, for instance, as early as 1833, a peculiar swelling met with in cattle, and known locally as *clyers*, had been somewhat carefully studied by Professor Dick. He dissected several animals which presented these tumors in the parts about the throat, and described them as having a somewhat malignant character, and corresponding to what were usually called "medullary carcinomatous tumors." He noticed, also, that sometimes abscesses developed, and that when these were laid open healing frequently followed, and that in other cases the open sores remained indolent for a time, and then increased until they interrupted respiration by their bulk, and prevented deglutition. In 1841, he made a further report on the same subject, describing now the mouth and throat of a cow in which there was a distinctive tumor, and he spoke of it as a mass of fungus flesh, while a part of the jaw was decayed and absorbed. This time he stated that the disease was not unknown in cattle, and that it was well known as attacking the jaws in human beings; that by surgeons it was demonstrated osteo-sarcoma of the maxillary bones, and he added that the remote cause seemed to be a scrofulous diathesis, while the existing cause was commonly dis-

1. Read at the Stated Meeting of the Buffalo Academy of Medicine, December 6, 1892.

ease of the molar teeth, or some accidental injury. In 1843, a Mr. Relph contributed to *The Veterinarian*, a "kind of indurated tumor," which he met with in his practice oftener than anything except the common wens. He spoke also of its ulceration and extension, until at length animals sank and died of "atrophy or phthisis pulmonarius." He spoke also of a section of one of these tumors as "mostly displaying several abscesses with matter varying in consistency, and often very fetid, enclosed in what seemed to be fibro-cartilaginous cysts." Also, again, of the implication of the nasal sinuses and involvement of the orbital cavity, where bone was being removed and matter deposited, and also that the tongue was much enlarged and ulcerated. In 1845, Professor Simmonds spoke of the diseased condition as an affection termed *scirrhus tongue*, and one frequently found associated with maladies essentially different. Under this term, scirrhus tongue, it was commonly described until quite recently. In 1864, a case was fully described in the *Ed. Vet. Rev.*, under the title of Cancer of the Tongue in an Ox. On the continent, also, this disease had been everywhere misunderstood. It had been frequently regarded as a tubercular infection or mistaken for a simple chronic glossitis; while the continental languages were full of common names for it, and veterinarians spoke of it as osteo-sarcoma, bone cancer, spina ventosa, etc. In 1876, Bollinger presented to the Society for Morphology and Philosophy, at Munich, a learned paper on the subject, with a demonstration of microscopical and gross specimens. He pointed out that these tumors consisted of several centers of growth, bound together by connective tissue, whose cut surfaces presented yellowish white suppurative foci, or at times a spongy texture, owing to the formation of minute cavities in a fibrinous crust, which contained a thick, yellow, caseous pulp. The microscope revealed a structure something like that of sarcoma, while the expressed juice contained, among other materials, small granular bodies which had a mulberry-like appearance, and were sometimes encrusted with chalky matter. He had discovered that these latter bodies were true fungi, and he further maintained from the constancy of their appearance that they were not accidental but had a pathological significance. These also he had met with in old museum specimens.

Bollinger had observed this remarkable formation not only in the upper and lower jaws, but also in the tongue, in which latter organ, when recent, it much resembled the condition produced by tuberculosis. If these nodules were situated on the surface of the tongue, they rapidly led to the formation of ulcers. It was possible, also, for an interstitial glossitis to occur, which, in spite of partial atrophy of muscle fibers, led to enlargement and peculiar hardness of the tongue, from which the infection gained, in German, its popular name, *holz-zunge*, or wooden-tongue. On continuing his researches further, he discovered the same fungus in tumors which occurred in the pharynx, larynx, and stomach. He even described this fungus in a case of so-called fibroid of the second stomach of a cow, and in a form of apparent tubercular ulceration of the intestines. He submitted the fungus to the botanist Harz, who described it and assigned it to a temporary position. Cultivation experiments and inoculation of the tongue of a calf with liquid containing the organism, all failed. Its name was given by Harz. Bollinger's paper was published in 1877, and attracted widespread attention, and brought out the claim from Perroncito and Rivolta that they had already discovered the same organism in 1863 and 1868 respectively. It is very probable that Hahn, of Munich, met with the fungus in 1870, since he states that in a case of wooden-tongue he found characteristic structures, which he described provisionally as a species of mould fungus, but Bollinger was certainly the first to study the organism systematically, and to throw an entirely new light upon the pathology of the infection, and his researches were corroborated by Siedamgrotzky, by Johne and Ponfick ; and so by one observer and another on the continent, and later in Great Britain, the pathological identity of the various lesions included under so many different names was established, and the names of Boström and Israel, of Axe and Crookshank, and of many others in addition to those already named, have become inseparable from the subject itself. During the year 1888, a most important memoir was sent to the Agricultural Department of the Privy Council by Professor Crookshank, of King's College, which was published in the annual report of that department for that year. It is a most elaborate document, extensively illustrated, and prepared with the greatest care, and to it the writer is largely indebted. One of the things for which we are especially indebted to Professor Crookshank in this matter, is the establishment of the fact that many of the lesions in animals which had before been considered due to tuberculosis, were in reality manifestations of actinomycotic disease, a beautiful proof of which that gentleman has kindly afforded me in his own laboratory, while in the document referred to it is amply evidenced by illustration. Altogether we regard this report pertaining to animals and men as well, and comprising eighty pages, as presenting, perhaps, the best summary of the subject which we have.

The disease, as Crookshank and others succinctly state, belongs to the class of infective granulomata. It produces an irritative, inflammatory action, by the presence of a special irritant, a microphyte, and consists of a collection of round, epitheloid, lymphoid, and giant cells, enclosed within a fibrous reticulum. There are thus constituted nodular tumors of various sizes. Sometimes these attain large dimensions, at other times they break down early and suppurate. While it appears, as seen in a former lecture, that the actinomycotic fungus may, by itself, produce pus, it is, nevertheless, equally true that suppuration in actinomycotic tumors is commonly due to a secondary infection by the ordinary pyogenic organisms. Calcification takes place sometimes in the fungus tufts, and altogether the actinomycotic nodule so closely resembles the tubercular in its minute character that it constitutes almost a mimicry of the latter. Its most characteristic manifestation in cattle is betokened by the popular name most frequently given to it, namely, *lumpy jaw*. It is especially prevalent in river valleys and marshes, and on land reclaimed from the sea, and in animals appears to occur more frequently in the winter, and more often in the young than in the old. There is strong reason for believing that the fungus gains access to the system through wounds or ulceration of the mucous membrane, or through carious tissue. It has been pointed out that the common occurrence of the disease at the time of second dentition may be due to the wounds produced during the shedding of the teeth. It is supposed, also, that thistles and frozen roots, by wounding the parts, may afford a path of entrance. Discharges from infected animals are thus infectious, and cow sheds and pastures, drinking troughs and fodder may be easily infected.

The fungus itself may be detected with the naked eye in the discharge of an actinomycotic ulcer, or in the scraping from a cut surface or growth. Its tufts vary in size from that of a grain of sand to that of a pin's head. If the material contained be spread out on a slide and examined against a dark back-ground, these granules appear white or yellow-white in color, but if examined in transmitted light they appear distinctly brownish. If they are pressed down with a cover-glass, they readily flatten out, while possibly a distinct gritty sensation is transmitted to the finger, owing to the presence of calcareous matter. With a low power the fungus will be recognized in the form of irregular patches scattered over the field, which might at first be mistaken for granular *débris*, but which on more exact examination will be observed to have a characteristic appearance. With a higher power masses will be seen which look like a rosette of clubs, or which have here and there throughout the margin a club-shaped projection. Of course, with these bodies, pus cells and other granules will be found. By pressing upon the cover, the rosette is broken up and then the club forms are recognized singly or in twos, or in the shape of fan-shaped segments. The presence of calcium salts may be readily demonstrated by the acids which dissolve them; by which the clubs are not affected. The club-shaped masses are not affected by ether, nor by potassium hydrate, which shows that they are not fat crystals. By teasing the little cluster we may break it up into its club-like elements, and these may be examined with still higher powers. When these grains are placed in water and teased out, the center portion seems to be composed of a structureless core. When the organism is removed from the disease in man, it appears to present minute and delicate filaments which are not present in those removed from animals.

Actinomyces were generally regarded as belonging to the higher micro-fungi until Boström put forward a new theory. By Harz and Johne the club-shaped prolongations were considered to be conidia and the threads to be hyphæ. Boström, on the other hand, thought that the clubs should not be regarded as conidia, but the result of a pathological stage of the threads, and he considered that the calcification which occurs in them supports his view. If this were the case, cultivations could not be successfully made from the clubs, but must be made from fungi containing threads. He made cultures from five animal cases and one human, and obtained a similar result from all. The fungi were isolated from pus with sterilized needles, were placed in liquified gelatine ; in which they were teased out, and the gelatine then spread on glass plates. In a few days the growth became visible. The fungi were then isolated from the plates, crushed between sterilized glass slides, and planted on the surface of nutrient agar and blood serum. In this way pure cultivations were obtained, and the culture, which I show you herewith, is made from a descendant of one of his original cultures. It grows in a peculiar way, with a finely granular whitish appearance, after a few days small yellowish-red spots appear in the center, increase in size for a week or so, and then become confluent, while the margin is also dotted with similar spots. Isolated colonies, as they appear, have a yellowish-red center, with a grayish margin. Boström states that the actinomycis is not one of the mould fungi, and its central threads do not constitute a mycelium. He was inclined to regard it as a branched cladothrix.

Johne and Ponfick made a careful series of experiments to prove that actinomycosis is transmissible from animal to animal. Inoculation experiments, whether subcutaneous or intraperitoneal, as well as intra-veinous injection, were successful. Feeding experiments gave negative results. It appears from their researches that rabbits and dogs possess a marked immunity, while the cow is, perhaps, the most susceptible of all animals.

We are, naturally, most interested in this disease as it appears in man. In 1878, Israel published the drawings made in 1845 by Langenbeck, then in Kiel, of a case of vertebral caries, in the pus from which peculiar bodies were observed. There can be but little doubt that these structures were the fungi of actinomycosis. But the first published observations will be found in Lebert's large work on pathological anatomy, described in the text and figured in the atlas. In 1848, Lebert received some pus of great consistency which had been obtained from an abscess in the thoracic wall of an elderly man. The patient had been thought to have a cancerous pulmonary affection. The pus contained a very considerable quantity of minute spherical beads, which could be crushed between two strips of glass, and in which, under a low power, radiating wedge-shape bodies were found. Lebert tested these bodies most carefully with chemical reagents, and had in mind the possible existence of some helminthic *débris* of which these bodies might be hooklets, but he sought in vain for the common parasites. Actinomycotic pus was later described and figured by Robin, but the first adequate description of the disease in man was made by Israel, who was soon followed by Ponfick. By these two writers the various cases which had been observed up to that date were collected and described and the disease classified according to the seat of invasion. Up to the time of Crookshank's report he had collected about one hundred and thirty human cases, and from those, as well as from the work of Israel, the following description is largely drawn:

*Invasion by the mouth and pharynx.*—The fungus may gain access through carious teeth or wounds or fistulæ of the jaw, and possibly by the pharynx and tonsils; it attacks the lower jaw most frequently. The consequent tumor is found in close connection with the bone, and just beneath the jaw or in front of the trachea. Actinomycotic tumors in this region appear to correspond very closely with the *clyers*, already described in cattle. As in cattle, they may discharge through the skin, although they differ in their tendency to form burrowing abscesses instead of recognizable tumors. The upper jaw is much less frequently attacked than the lower. The progress of the disease is usually slow, and there is a tendency to the deep-seated parts becoming involved; while, when the lower jaw is attacked, the tumors tend to approach the surface. In other cases the disease has been described as extending from the aveolar process to the temporal bone, or the base of the skull, destroying these bones and even reaching the brain. In yet other cases, it has extended along the spinal column, implicating the vertebræ and traveling and pointing in various directions. The first cases of actinomycosis hominis which were observed in this country, were connected with the jaw, and were described by Dr. Murphy, of Chicago. Both recovered after thorough curetting. In one recorded case the disease existed for seven years, was localized in the bronchi (bronchitis actinomycotica), and did not extend into the lungs. The sputum was examined and contained the characteristic fungus. If the fungi be inhaled, they pass into the lungs and produce proliferation of round cells, which undergo fatty degeneration. The resulting patches of peri-bronchitis, or pneumonia, become yellowish white. Suppuration and hemorrhage follow and the resulting small cavities contain pus cells, fat granules, blood and fungi; ultimately a dense layer of connective tissue is formed around the cavities, which are lined with granulation tissue containing the characteristic fungus. The symptoms are usually obscure, but the specific organism may be found in the sputum, and is, in fact, often recognizable with the naked eye. The apices of the lungs are not, as a rule, affected. There is considerable resemblance in clinical course to chronic or fibroid phthisis. In the second stage the symptoms are more severe. The disease spreads to neighboring parts and often pleurisy supervenes. The disease has been known to descend behind the diaphragm and point as a psoas or lumbar abscess; or to perforate the diaphragm, reach the abdominal cavity, and there set up peritonitis or subphrenic abscess. The disease may also extend forward in the direction of the pericardium and anterior mediastinum. In the third stage the disease comes to the surface, either over the chest or in the neighborhood of the vertebræ; a livid swelling appears, from which no fluid escapes on puncture, but which works its way to the surface, and then discharges the muco-purulent fluid, in which the fungi are easily recognized.

*Invasion by the digestive tract.*—Chiari has published a case dying from general marasmus, after two years' illness, at the age of thirty-four. The mucous membrane of the intestines was completely covered with whitish patches closely adherent to the adjacent tissues. In this case the teeth were carious. In such cases, generally, small nodules about the size of a pea may be found in the sub-mucous tissue and in the mucosa itself, they soften and form ulcers with determined edges, their bases reaching the deepest layer. These may undergo cicatrization, but, generally, ulceration extends through the peritoneum to the abdominal cavity, and we have perforation of the bladder or the intestines, or perhaps of the abdominal wall. Symptoms are absent or not characteristic. The fungus may be found in the evacuations, or by exploratory puncture.

Besides such cases as the above, which can be easily classified, there are a number of recorded cases presenting varied symptoms and anatomical relations in which the path of infection has not been determined. It may be said in common of all cases, however, that no matter where the lesions may be situated, the discharges therefrom present always typical characteristics.

The first case of actinomycosis in man met with in Great Britain was described by Dr. Harley in 1885, the patient being an inmate of St. Thomas' Hospital. The next two cases were recognized by Mr. Shattock, who published them in the Pathological Transactions in 1884 and 1885. Numerous other cases are described in English journals, and it is a matter of interest to know that numerous specimens which had long been deposited in museums, that of the College of Surgeons, for instance, and had been described under other names, were found to be genuine cases of actinomycosis. It is worth while, also, to know that since the introduction of Gram's method of staining, a distinctive advance in the recognition of the organism has been made, since by this method, combined with staining by orange rubin, as pointed out by Crookshank, the threads are stained blue and the clubs crimson, while in the younger clubs the protoplasm of the thread can be traced into the interior of the club. Evidently, then, the best method of preparation is Gram's stain, followed by eosin or orange rubin. Inasmuch as Gram's method has not been applied by either Israel or Koch in the earlier part of their studies of this disease, it is not strange that most of their writings have failed in the complete description of the organism which the later reporters have given.

So far as the source of the disease in man is known, many interesting observations have been made. Two cases have been recorded in support of a theory of direct infection from the cow. One of these occurred in a man who had the care of animals, some of which were diseased, the other in a man who had the charge of cows, one of which had a tumor of the jaw, which tumor he had opened. On the other hand, Moosbrugger found that out of seventy-five cases, fifty-four were in men, nineteen in women, and two in young girls. In eleven of these the occupation was not stated; in thirty-three their occupations did not bring them into contact with diseased animals. Only ten of his cases occurred among farmers, peasants, and farm laborers, and only one of these ten had been brought into contact with diseased animals. Of the twenty-one women there were only four peasants, and none of them had been taking care of diseased animals. Infection by the flesh of diseased animals has also been discussed, but there is no evidence of prevalence of the disease among butchers, who would be particularly liable to it if flesh were a source of infection. Moreover, the chances of infection from this source are minimized by the flesh being almost always cooked. To be sure, the disease occurs also in pigs, and pork is often eaten in an uncooked state; and Israel has pointed out that the disease has never been known to occur among strict Jews. Evidence seems to point to the origin of the disease among the cereal foods. This view is supported by observations with reference to the part played by cereals in inducing the disease in cattle, an origin well known, and it gains additional support from a case described by Soltman where the disease resulted from swallowing an awn of barley. This was accidentally swallowed by a boy of eleven, who became very ill and suffered great pain behind the sternum, extending to the back. An abscess formed, covering an area extending over this inter-costal space, and when opened the awn of barley was found in the evacuated pus, but pain continued and fresh deposits occurred, and when the boy was taken to the hospital, the specific fungus was detected.

As Crookshank states, it has not been possible to trace every step in the life history of many of the basidiomycetes; but if we regard the ray fungus in the light of what is known to occur in many species, this life history may be explained about as he explains it, thus: The spores sprout into hyphæ, and these form fine threads which branch irregularly and sometimes dichotomously; the extremities of the branches develop the club-shaped bodies; these are so closely packed together that a more or less globular body is formed with a central core, composed of a dense mycelium. By Gram's method, these threads can be differentiated into an external sheath with protoplasmic contents. The club-shaped body appears to be externally mucilaginous, while internally it is continuous with the protoplasm of the thread. In all probability the clubs represent organisms of fructification. If this be correct, the protoplasm in the interior of the club may possibly undergo changes leading to spore formation, the spores being ultimately set free. At other times, the clubs themselves seem to sprout, and the sprouting forms to suggest teleuto spores. In whatever way formed, there is but little doubt that spores are set free in the vicinity of a rosette, and give rise to fresh individuals, and that spores and young fungi are taken up by wandering cells, by which they are conveyed to a distance where they reproduce their kind.

At the Congress of German Surgeons in 1890, Israel and Wolff made an important communication on ray fungi, and their successful cultivation outside of the animal organism, and their successful implantation of such cultures upon the animal. In this a claim was made that they were the first to succeed in cultivating the actinomycosis and implanting it, which claim would appear to be unjustifiable, since, so far as I can learn, Boström anticipated them in this work by several years. Still, their remarks and their demonstrations were most entertaining, and the question of priority is one of minor importance. That they did succeed is established, and the fact that it is impossible to produce an artificial actinomycotic disease in animals is established beyond a doubt.

The cultures which I show you here were made from some furnished me by Dr. Karg, of Leipsic, who received his directly from Boström.

At the Congress of German Surgeons, held in Berlin, (*Prager medicinische Wochenschrift*, July 27, 1892,) Schlange reported observations of 130 cases of actinomycosis. Over one-half of these were traced since 1886, and their history warrants a more favorable prognosis than has hitherto been attached to the disease.

In eighty of these cases, the process was limited to the head and neck; forty have been cured for over two years. One case, in which the disease progressed along the base of the skull, died in six months from the inception, in consequence of meningitis. Another of these cases, which developed downwards, is still alive, thirteen years after beginning of the affection.

Regarding treatment, the author calls attention to the fact that extensive extirpations have been abandoned. Surgeons now content themselves with small incisions, scooping, etc., because actinomycosis tends to spontaneous recovery. He adds that it is barely credible how widespread an actinomycosis may recover without treatment of any kind. Even actinomycosis of the lungs is amenable to healing, except when the thoracic parietes are much perforated by the ray fungus. Even actinomycosis involving the abdominal organs is not excluded from possible recovery. When it affects the intestines, the prognosis is bad; but when located in the abdominal walls the prognosis is more favorable.

Billroth's suggestion to treat actinomycosis with Koch's tuberculine, has not proven efficacious.

Garré agrees that actinomycosis admits of a more favorable prognosis than has been given it. Cases which appeared hopeless recovered in five to six years. Of the twenty cases he observed, but two terminated fatally. He thinks that most cases of actinomycosis are dependent upon a mixed infection. He further believes that the anerobic fungi die on exposure to the air.

v. Eiselsberg, at the same Congress, mentioned a case of extensive actinomycosis of the abdominal parietes, which was cured in one year by ten injections of Koch's lymph. He described another case, in Kahler's Clinic, in Vienna, now being treated with Kochin, which is approaching recovery.

The first case of actinomycosis of the liver observed in this country occurred in the practice of Kneer, of New York, 1879. He made a number of most perfect microscopic slides of the specimen, which he presented to many investigators.

C. K., aged about thirty, was seen by me with Dr. F., June 18, 1892. I was told that he had enlargement of the liver, with a peculiar abscess, discharging externally, and that there was suspicion of malignant growth.

With great difficulty I secured even the following meager history:

Some two and a half years ago he spent a winter at the stock yards, clerking and assisting in office work in the main, although still coming in contact with cattle. At that time he was strong and robust; since that time he has never been well. He told me, in answer to questions, that he had seen two or three cases of lumpy-jaw in cattle. I learned that during that season he began to cough, and that from that time he has been more or less troubled with a constant cough. Some months later he began to complain of vague and indefinite pains on the right side. After ceasing to work at the stock-yards he became a clerk in the City Hall, but for the past year has coughed and raised more or less. At one time he was quite an athlete, but his companions noticed, during the Summer of 1891, that he was not as strong, and that he seemed greatly exhausted after even comparatively violent exercise. During the past Winter, he became paler and somewhat emaciated. He went to Dr. B. and showed him a lump in the right side, apparently involving the ribs over the liver, and in the axillary line. For some reason, Dr. B. told him that he had broken one or more ribs, although there was no history of violence. I suppose he regarded the lump, of which the patient complained, as callus. Some time after this, and about four months ago, he first went to Dr. F., who detected pseudo-fluctuation in this mass, and made an incision. A quantity of material was discharged, which did not look like ordinary pus, but seemed a sort of *débris*. About this time, the young man took to the house, and later to the bed, complaining of constantly increasing pain and soreness in the region of the liver and lower part of the right lung. At various times different spots in the area above alluded to would soften apparently as when suppurating, acting much like small abscesses, and would be incised, discharging material of the same character as before. In different directions, a probe would pass to a depth of from two to four inches. He was in this general condition, growing steadily worse, when I first saw him. At this time, he moved in bed with difficulty, was very pale and emaciated, pulse weak, sweating easily, and looked much like one in an advanced stage of consumption. On the right side, over the lower ribs, was a brawny, livid swelling, with several sinuses, into which the probe passed to different depths. The patient was coughing frequently, and raising sputum, much like that of consumption. The doctor had just incised one of the little areas, and in the sanguineous fluid, which still escaped, were little yellow particles, like minute particles of cheese. There was an extensive area of dulness, the liver seeming much enlarged, and either it was enlarged upward or the lower portion of the lung was consolidated; still dulness, upon percussion, extended almost up to the line of the nipples. Specimens of the sputum and of the discharge were secured for microscopical examination.

At that time, I made a differential diagnosis of actinomycosis, for these reasons: The general appearance indicated either malignant disease or one of the infectious granulomata. The former I could exclude, I thought, because of the long history of the case, the absence of jaundice and of ascites, also on the ground of the family history and the age of the patient. It seemed to me clearly to belong among the infectious granulomata. There was no history nor any evidence of syphilis. The trouble seemed to me too extensive and locally comprehensive for manifestation of tuberculosis, and I was practically narrowed down by exclusion to the question of actinomycosis. There was nothing in the history nor the general appearance of the patient to preclude this, while the characteristic minute yellow particles in the discharge from one incision made me feel quite positive.

On returning and placing the specimens under the microscope, the diagnosis was at once confirmed. The sputum contained no tubercule bacilli, but an enormous number of actinomycotic fungi, while the same were in the discharge from the sinus. The patient was in bad, general condition; but holding that if his life were to be saved at all, it could only be by extreme measures, I suggested operation as the only possible measure for relief. This was made June 25th, by Dr. F., Dr. H. U. Williams and myself being present by his courtesy. It consisted of a resection of the three ribs in the center of the mass most involved. It was apparent then that an enormous thickening of the overlying tissues outside of the bony wall had occurred, and that much of the enlargement which we had credited to his liver was really due to this thickening, we having been deceived partly because of his extreme tenderness in this region. A large portion of sloughing and infectious tissue was cut away, along with the ribs, and the sharp spoon was used very freely, but there seemed to be no limit to the infected mass, and impending collapse suggested the termination of the operation long before desirable limits had been reached. The patient died, the same day, of shock.

In all the tissue removed, as well as in such fluid as could be expressed from the same, and from the bone marrow of the excised portion of rib, myriads of fungi were detected, while culture experiments gave impure cultures of actinomycotic fungi, the same being mixed with the ordinary pyogenic organisms. I am indebted to Dr. Williams for much of the microscopical examination of the specimens from this case.

510 Delaware Avenue.

